# Effect of Fear of Pain on Lumbar–Pelvic and Trunk–Pelvic Kinematics in Individuals With Chronic Non‐Specific Low Back Pain: A Cross‐Sectional Study

**DOI:** 10.1002/hsr2.73398

**Published:** 2026-11-10

**Authors:** Mohammad Reza Salehifar, Rozita Hedayati, Marzieh Mortezanejad, Rasool Bagheri, Fatemeh Paknazar

**Affiliations:** ^1^ Neuromuscular Rehabilitation Research Center, Neuroscience Research Institute Semnan University of Medical Sciences Semnan Iran; ^2^ Social Determinants of Health Research Center Semnan University of Medical Sciences Semnan Iran

**Keywords:** balance control, chronic non‐specific low back pain, fear of pain, lumbopelvic coordination

## Abstract

**Background:**

Impaired trunk movement associated with low back pain and fear of pain may reduce range of motion and disrupt lumbopelvic coordination in individuals with chronic non‐specific low back pain (CNSLBP).

**Aim:**

To investigate the association between fear of pain and trunk–pelvic and lumbar–pelvic kinematics under different perturbation levels and visual conditions in individuals with CNSLBP.

**Methods:**

This cross‐sectional study included 50 individuals with CNSLBP, who were categorized into high fear of pain (*n* = 24) and low fear of pain (*n* = 26) groups based on the Pain Anxiety Symptoms Scale (PASS‐20). A Biodex Balance System SD integrated with a Qualisys motion analysis system was used to generate postural perturbations while simultaneously capturing kinematic data.

**Results:**

After adjustment for age, the primary mixed‐model analysis revealed significantly greater trunk–pelvic angular displacement (*p* < 0.001) and significantly greater trunk–pelvic angular velocity in the high fear‐of‐pain group than in the low fear‐of‐pain group (*p* = 0.014). Exploratory analyses further indicated greater trunk–pelvic and lumbar–pelvic angular displacement in the transverse plane during eyes‐open conditions on unstable surfaces among participants with high fear of pain (*p* < 0.05). No significant between‐group differences were observed under eyes‐closed conditions.

**Conclusion:**

Higher levels of fear of pain may be associated with altered postural control in individuals with CNSLBP, particularly during visually guided balance tasks on unstable surfaces. After adjustment for age, between‐group differences were primarily observed in transverse‐plane trunk–pelvic and lumbar–pelvic motion, suggesting a potential role for pain‐related fear in modulating rotational movement control.

## 1 Introduction

Low back pain (LBP) is one of the most common musculoskeletal conditions, with a prevalence of 84% among adults [1]. Approximately 5% of affected individuals develop chronic LBP [2]. Patients with chronic LBP account for 73%–77% of all LBP cases, of which approximately 85% are classified as nonspecific and are therefore referred to as chronic non‐specific low back pain (CNSLBP) [3].

The etiology of CNSLBP is widely considered multifactorial, involving a complex interplay between mechanical alterations and psychosocial distress [2, 4, 5]. Biomechanical factors are often associated with repetitive and prolonged spinal loading, which may compromise neuromuscular control and contribute to persistent pain [6]. Specifically, alterations in spinal and lower‐limb dynamics—such as joint rigidity, muscle stiffness, and delayed muscle activation—may result in asymmetrical mechanical loading of the lumbar spine [7, 8]. These alterations in motor activity and control strategies are not limited to static postures; recent evidence indicates that kinematic deviations in CNSLBP are also observed during various functional tasks, including gait and segmental coordination [9, 10, 11, 12].

Crucially, these altered movement patterns are strongly influenced by postural control deficits associated with CNSLBP. Individuals with CNSLBP may have difficulty adapting efficiently to multidirectional postural challenges, often exhibiting an overly rigid co‐contraction strategy or delayed reflexive responses [13]. To evaluate these deficits, researchers use perturbation‐based tasks and visual manipulations (e.g., eyes open vs*.* eyes closed), as these challenges require the central nervous system to reweight sensory inputs from somatosensory, visual, and vestibular systems [14]. While earlier studies, such as that by Karayannis et al., established a broad association between psychological distress and altered trunk reflexes [6], they have often provided limited insight into how these alterations manifest as segment‐specific kinematic adaptations when the postural system is progressively challenged under unstable conditions.

Psychosocial factors, particularly fear of pain, may further contribute to these motor deficits through avoidance behaviors and protective movement strategies [15, 16, 17]. Fear of pain may disrupt the automaticity of postural control, possibly because pain‐related anxiety competes with balance regulation for limited information‐processing resources [18]. This interpretation is also supported by neurophysiological evidence demonstrating connections between emotional‐processing regions, including the amygdala and limbic system, and brainstem structures involved in vestibular and postural control [19, 20, 21].

Despite these established connections, an important gap remains in the literature. Although fear‐avoidance behavior is known to influence movement, it remains unclear how different levels of fear of pain affect segment‐specific kinematics—particularly trunk–pelvic and lumbar–pelvic coordination—under graded mechanical perturbations and different visual conditions. Most existing studies have focused on either psychological correlates or basic static balance tasks [22, 23, 24], leaving the interaction among perturbation intensity, visual manipulation, and plane‐specific kinematic behavior insufficiently explored.

To address this gap, this study aimed to investigate the effects of high versus low fear of pain on trunk–pelvic and lumbar–pelvic kinematics in individuals with CNSLBP across different perturbation intensities and visual conditions (eyes open vs*.* eyes closed). We hypothesized that:

1. Individuals with CNSLBP and high fear of pain would exhibit significantly smaller maximum angular displacement in the sagittal and transverse planes during dynamic perturbations than those with low fear of pain, reflecting an overprotective and rigid movement strategy.
2. Conversely, in the frontal plane, individuals with high fear of pain would demonstrate greater or less well‐controlled angular displacement under high‐intensity perturbations and eyes‐closed conditions, reflecting a reduced ability to maintain lateral lumbopelvic stability when automatic postural responses are compromised by fear.

## 2 Materials and Methods

### 2.1 Study Design

This study employed a cross‐sectional design and was approved by the Ethics Committee of Semnan University of Medical Sciences. The outcome measures included maximum angular displacement and angular velocity of the trunk–pelvic and lumbar–pelvic segments across three balance conditions (Level 2, Level 5, and static) and three kinematic planes (sagittal, frontal, and transverse).

### 2.2 Sample Size

An initial sample‐size estimation was performed using G*Power based on a pilot study (*n* = 10 per group). For the primary outcome of this study (trunk–pelvic angular displacement velocity in the mixed model), the pilot data suggested a large effect size (Cohen's *d* ≈ 0.90). Because effect‐size estimates derived from small pilot samples may be unstable and may overestimate the true population effect, we adopted a more conservative effect size of 0.60 for the primary sample‐size calculation. With *α* = 0.05 and 80% power (two‐tailed), this yielded a required sample size of 25 participants per group. The study initially aimed to recruit at least 25 participants in each group and recruited 27 participants per group to allow for potential exclusions and withdrawals. After application of the predefined eligibility criteria and accounting for participant withdrawals/non‐compliance and practical recruitment constraints, the final analyzable sample consisted of 24 participants in the high‐fear group and 26 participants in the low‐fear group (total *N* = 50).

### 2.3 Subjects

Participants were recruited from clinics, hospitals, and physiotherapy centers using convenience sampling. The inclusion criteria were: (i) a diagnosis of chronic non‐specific low back pain (CNSLBP) persisting for at least 12 months before testing, (ii) age between 20 and 55 years [25], and (iii) low pain intensity, defined as a visual analog scale (VAS) score ≤ 3 [26]. This threshold was intentionally selected based on biomechanical and safety considerations specific to the experimental design.

The exclusion criteria included severe spinal pathology, cauda equina syndrome, sciatica, spinal canal stenosis, nerve root involvement, previous spinal surgery, scoliosis or kyphosis, radicular pain extending to the gluteal fold, situational dizziness, spinal fracture, joint swelling or inflammation, uncorrected visual impairment, learning disability, pregnancy, and cognitive or memory impairment [25]. After familiarization with the experimental procedures, participants provided written informed consent. They then rated their pain intensity using the VAS and completed the Pain Anxiety Symptoms Scale (PASS‐20) to assess fear of pain. Based on PASS‐20 scores, participants were categorized into two groups: high fear of pain (≥ 30) and low fear of pain (< 30) [27].

### 2.4 Experimental Procedure

Participants first completed the informed consent form, VAS, and PASS‐20 questionnaire. Kinematic data were then collected using a Biodex Balance System (Biodex Medical Systems, Shirley, NY, USA; Model SD 950–302) integrated with a Qualisys motion analysis system (Qualisys AB, Göteborg, Sweden).

Reflective markers were placed on predefined anatomical landmarks of the trunk, lumbar, and pelvic regions. Foot placement was standardized and recorded on the Biodex platform to ensure consistent positioning and stability conditions. Participants were familiarized with the experimental setup before data acquisition.

Motion capture using the Qualisys system was initiated simultaneously with the Biodex Balance System, and each trial lasted 20 s.

In the first trial, participants performed single‐leg standing with eyes open under the static Biodex condition. This procedure was used to reduce the base of support and increase postural demand at baseline, thereby enhancing sensitivity to subtle impairments in sensorimotor control and fear‐related adaptations in lumbopelvic kinematics.

Subsequently, participants performed double‐leg standing trials under eyes‐open and eyes‐closed conditions at Biodex stability levels 5 and 2 in randomized order. Kinematic data were recorded under all experimental conditions, including static, Level 5, and Level 2 perturbations.

To minimize potential confounding effects, such as motor learning and fatigue, the order of the experimental conditions involving visual and perturbation manipulations was randomized for each participant using a computer‐generated block randomization sequence.

### 2.5 PASS‐20 Questionnaire

Pain‐related anxiety was assessed using the PASS‐20. The PASS‐20 measures pain‐specific anxiety symptoms and consists of four subscales: cognitive anxiety, escape/avoidance behaviors, fear of pain, and physiological symptoms of anxiety, comprising a total of 20 items. Each item is rated on a frequency scale from 0 (never) to 5 (always). The total score ranges from 0 to 100, with higher scores indicating greater pain‐related anxiety. As described above, the PASS‐20 subscales have demonstrated satisfactory internal consistency, test–retest reliability, and validity [28]. The Persian version of the PASS‐20 has acceptable psychometric properties for assessing pain‐related anxiety in Persian‐speaking patients with chronic LBP [29]. Based on total PASS‐20 scores, participants were categorized into two groups: high fear of pain (≥ 30) and low fear of pain (< 30) [27].

### 2.6 Instrumentation (Biodex Balance System and Motion Analysis System)

#### 2.6.1 Biodex Balance System

Participants were positioned on a Biodex Balance System (Biodex Medical Systems, Shirley, NY, USA; Model SD 950–302) under three stability conditions: maximum stability (static), moderate instability (Level 5 of 12), and high instability (Level 2 of 12). Each participant was initially aligned on the Biodex platform. The Biodex Balance System incorporates a dynamic platform with adjustable stability levels ranging from Level 12 (most stable/rigid) to Level 1 (least stable/most unstable), with progressively decreasing platform resistance as the level number decreases [30].

Foot placement was standardized by identifying and maintaining optimal heel and second‐toe positions throughout all trials to ensure consistent alignment and balanced weight distribution. Three familiarization trials were completed before data collection to minimize learning effects.

In the static condition, participants initially performed single‐leg standing with eyes open during the first trial. They subsequently performed double‐leg standing under both eyes‐open and eyes‐closed conditions at Biodex instability levels 5 and 2. Each participant completed five experimental conditions: static eyes open, Level 2 eyes open, Level 2 eyes closed, Level 5 eyes open, and Level 5 eyes closed. The static eyes‐open condition was not randomized because it was predefined as the baseline measurement and was therefore always performed first. Following this baseline trial, the remaining experimental conditions (Biodex level × eye condition) were randomized for each participant. Each trial lasted 20 s, with a 5‐min rest period between conditions to minimize fatigue effects [31].

#### 2.6.2 Motion Analysis System

The Biodex Balance System was embedded within a motion‐analysis environment consisting of a Qualisys motion analysis system equipped with Qualisys Track Manager (QTM) software (Qualisys AB, Göteborg, Sweden). Participants stood barefoot on the Biodex platform while the Qualisys system was used to record kinematic measurements. The test environment was first calibrated using an l‐frame and T‐wand. Ten reflective markers were attached to predefined anatomical landmarks in the trunk, lumbar, and pelvic segments. The trunk segment was defined using markers placed on both acromions and the T12 spinous process; the lumbar segment was defined using markers on the T12 spinous and transverse processes and the second sacral vertebra (S2); and the pelvic segment was defined using markers on S2 and the bilateral anterior superior iliac spines (ASIS) and posterior superior iliac spines (PSIS) (Figure 1) [32].

**Figure 1:**
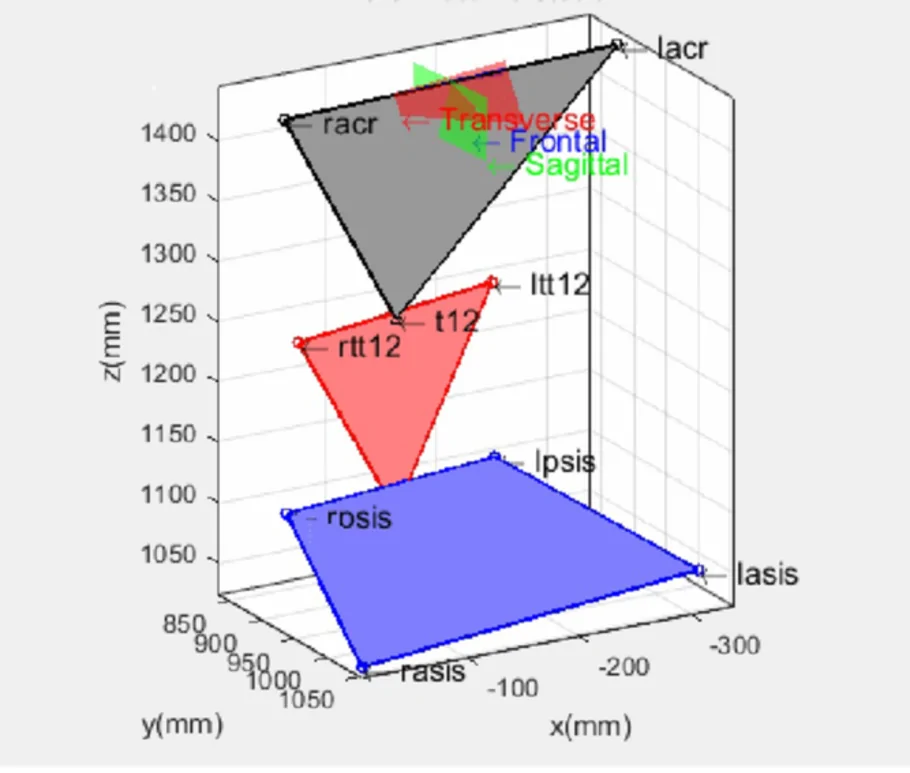
Schematic shape of adhesive markers. Abbreviations: LACR, left acromion; LASIS, left ASIS; LPSIS, left PSIS; RACR, right acromion; RASIS, right ASIS; RPSIS, right PSIS; LTT, left transverse process T12; RTT, right transverse process T12.

### 2.7 Kinematic Data Processing

This study quantified the maximum angular displacement and angular velocity of the lumbar and trunk segments relative to the pelvic segment in three anatomical planes: sagittal, frontal, and transverse. Kinematic data were collected using Qualisys Track Manager (QTM), which exported marker trajectories in tab‐separated value (TSV) format. A biomechanical model was then used to define marker coordinates within a global XYZ reference system corresponding to the three anatomical planes, allowing reconstruction of trunk and lumbar segment motion.

Maximum angular displacement of the trunk and lumbar segments relative to the pelvic segment was calculated for each kinematic plane and across three balance conditions (static, Level 2, and Level 5). These calculations were performed using MATLAB. For each 20‐second trial, maximum angular displacement was defined as the peak deviation observed over the full data‐acquisition period.

Finally, maximum angular displacement values for each segment and condition (static, Level 5, and Level 2) were normalized to 100% of the full range between the minimum and maximum stability conditions to allow standardized comparisons across participants and conditions.

### 2.8 Statistical Methods

SPSS version 26 was used for statistical analysis. The normality of the data was assessed using the Kolmogorov–Smirnov test. Based on the normality test results, Student's *t*‐test, the Mann–Whitney U test, and the *χ* ^2^ test were used to compare demographic variables between the groups.

The dependent variables—trunk–pelvic angular displacement and velocity and lumbar–pelvic angular displacement and velocity—were analyzed using the primary mixed‐model analysis described below. The significance level was set at 0.05.

#### 2.8.1 Primary Analysis

The primary hypothesis was tested using a mixed‐model analysis (Table 2), with fear group (high vs. low) as a fixed factor, age as a covariate, and Biodex level, kinematic plane, and eye condition as repeated factors. The dependent variables were angular displacement and angular displacement velocity of the trunk–pelvic and lumbar–pelvic segments.

#### 2.8.2 Exploratory Analyses

Stratified comparisons across individual Biodex levels, kinematic planes, and eye conditions (presented in Tables 3A, 3B, 4A, and 4B) were performed using independent *t*‐tests or Mann–Whitney U tests, as appropriate. These analyses were not corrected for multiple comparisons and were considered hypothesis‐generating; therefore, the results should be interpreted cautiously.

Reliability was assessed both within and between testing sessions. Intra‐session reliability was evaluated using repeated trials conducted during a single session, whereas inter‐session reliability was assessed in a subsample of 10 participants (5 from each group) who repeated the testing protocol 24 h after the initial assessment. Intraclass correlation coefficients (ICCs) were calculated for both analyses.

## 3 Results

The results are presented as follows: Table 1 shows the demographic comparisons, Table 2 presents the primary mixed‐model analysis, and Tables 3A, 3B, 4A, and 4B report the exploratory stratified analyses.

**Table 1:** Participant characteristics according to the fear‐of‐pain group.

Variable	High fear of pain (*n* = 24)	Low fear of pain (*n* = 26)	*p* value
Age (years)	45.00 ± 6.26	36.26 ± 8.99	< 0.001^a^
BMI (kg/m^2^)	28.63 ± 5.14	26.41 ± 5.01	0.129^a^
Male, *n* (%)	9 (37.5)	13 (50.0)	0.374^b^
Female, *n* (%)	15 (62.5)	13 (50.0)	

*Note:* Values are presented as mean ± SD or *n* (%).

^a^ Independent *t*‐test.

^b^ *χ* ^2^ test.

**Table 2:** Estimated marginal means of angular displacement and angular velocity according to the fear‐of‐pain group.

Outcome	High fear of pain Mean (95% CI)	Low fear of pain Mean (95% CI)	*p* value	*ηp* ^2^
Trunk‐pelvic angular displacement (°)	35.78 (35.01–36.55)	31.75 (31.02–32.48)	< 0.001	0.052
Lumbar‐pelvic angular displacement (°)	45.27 (44.44–46.10)	44.05 (43.25–44.85)	0.052	0.004
Trunk‐pelvic angular velocity (°/s)	64.90 (57.56–72.25)	51.23 (44.22–58.25)	0.014	0.007
Lumbar‐pelvic angular velocity (°/s)	83.59 (74.36–92.83)	73.57 (64.75–82.40)	0.150	0.002

*Note:* Adjusted using Linear Mixed Models with age as a covariate, and groups, kinematic plane, Biodex level, and eye condition as fixed factors. CI = confidence interval. *ηp* ^2^ values were calculated from *F*‐statistics and degrees of freedom using the formula *ηp* ^2 ^= (*F *× df_effect)/(F × df_effect + df_error). Age was a significant covariate only for trunk‐pelvic angular displacement (*β* = −0.116, SE = 0.033, *p* < 0.001).

**Table 3A:** Trunk–pelvic angular displacement according to Biodex level, kinematic plane, visual condition, and fear‐of‐pain group.

Biodex	Plane	Eye condition	High fear (mean ± SD)	Low fear (mean ± SD)	*p* value (unadjusted)	*p* value (adjusted)	Cohen's *d* for unadjusted age (95% CI)	Partial eta squared for adjusted age
Level 5	Sagittal	Open	81.71 ± 7.61	77.24 ± 7.00	0.077	0.059	−0.47 (−1.03, 0.09)	0.084
Level 5	Sagittal	Closed	81.76 ± 9.06	79.42 ± 8.25	0.145	0.292	−0.25 (−0.81, 0.31)	0.0246
Level 5	Transverse	Open	8.35 ± 4.08	7.52 ± 4.55	0.415	0.005*	−0.62 (−1.18, −0.05)	0.165
Level 5	Transverse	Closed	6.44 ± 3.40	6.62 ± 5.36	0.547	0.583	−0.24 (−0.79, 0.32)	0.0067
Level 5	Frontal	Open	23.24 ± 13.63	13.67 ± 8.34	0.005*	0.184	−0.63 (−1.19, −0.06)	0.039
Level 5	Frontal	Closed	15.03 ± 9.41	13.15 ± 7.65	0.587	0.984	−0.15 (−0.70, 0.41)	0.000
Level 2	Sagittal	Open	82.12 ± 6.58	77.17 ± 6.73	0.010*	0.093	0.45 (−0.41, 0.70)	0.063
Level 2	Sagittal	Closed	82.75 ± 7.23	78.74 ± 7.03	0.060	0.262	−0.35 (−0.91, 0.21)	0.028
Level 2	Transverse	Open	7.81 ± 3.68	7.02 ± 3.80	0.393	0.009*	−0.59 (−1.16, −0.02)	0.141
Level 2	Transverse	Closed	7.24 ± 5.88	6.73 ± 4.33	0.923	0.568	−0.39 (−0.95, 0.17)	0.007
Level 2	Frontal	Open	24.69 ± 13.63	13.79 ± 7.72	0.002*	0.338	−0.68 (−0.94, 0.18)	0.020
Level 2	Frontal	Closed	17.97 ± 11.54	14.72 ± 8.59	0.437	0.405	0.10 (−0.46, 0.65)	0.015
Static	Sagittal	Open	81.47 ± 7.19	78.50 ± 7.48	0.091	0.161	−0.38 (−0.93, 0.19)	0.040
Static	Transverse	Open	6.23 ± 7.07	3.71 ± 2.97	0.041*	0.108	−0.57 (−1.13, 0.00)	0.056
Static	Frontal	Open	10.01 ± 5.82	10.03 ± 7.07	0.846	0.131	−0.54 (−1.11, 0.03)	0.049

*Note:* Values are presented as mean ± SD. Adjusted *p* values were obtained from ANCOVA controlling for age. Cohen's *d* values are reported with 95% confidence intervals. *p* < 0.05 was considered statistically significant.*significancy, *p < *0.05.

**Table 3B:** Lumbar–pelvic angular displacement according to Biodex level, kinematic plane, visual condition, and fear‐of‐pain group.

Biodex	Plane	Eye condition	High fear (mean ± SD)	Low fear (mean ± SD)	*p* value (unadjusted)	*p* value (adjusted)	Cohen's *d* for unadjusted age (95% CI)	Partial eta squared for adjusted age
Level 5	Sagittal	Open	107.20 ± 7.99	107.47 ± 6.67	0.741	0.941	0.12 (−0.44, 0.67)	0.000
Level 5	Sagittal	Closed	108.06 ± 13.53	110.54 ± 5.50	0.756	0.512	0.30 (−0.26, 0.86)	0.010
Level 5	Transverse	Open	10.71 ± 5.26	10.71 ± 5.85	0.907	0.007*	−0.56 (−1.12, 0.01)	0.150
Level 5	Transverse	Closed	8.63 ± 4.41	9.15 ± 6.74	0.712	0.880	−0.19 (−0.74, 0.37)	0.001
Level 5	Frontal	Open	22.13 ± 15.10	16.04 ± 7.44	0.313	0.585	−0.39 (−0.95, 0.17)	0.007
Level 5	Frontal	Closed	16.85 ± 10.24	14.05 ± 6.00	0.534	0.362	0.24 (−0.41, 0.70)	0.018
Level 2	Sagittal	Open	108.16 ± 6.41	107.94 ± 6.46	0.641	0.744	0.13 (−0.43, 0.68)	0.002
Level 2	Sagittal	Closed	110.62 ± 6.94	110.37 ± 6.08	0.816	0.963	0.10 (−0.45, 0.66)	0.000
Level 2	Transverse	Open	10.36 ± 5.09	9.90 ± 5.03	0.669	0.020*	−0.50 (−1.07, 0.06)	0.115
Level 2	Transverse	Closed	9.75 ± 7.27	9.47 ± 5.82	0.938	0.668	−0.36 (−0.91, 0.21)	0.004
Level 2	Frontal	Open	25.74 ± 14.25	15.97 ± 7.82	0.015*	0.474	−0.42 (−0.87, 0.24)	0.011
Level 2	Frontal	Closed	19.53 ± 14.10	16.41 ± 8.03	> 0.999	0.364	0.16 (−0.40, 0.71)	0.020
Static	Sagittal	Open	109.60 ± 9.42	110.23 ± 5.65	0.786	0.602	0.22 (−0.34, 0.77)	0.006
Static	Transverse	Open	8.38 ± 8.65	5.26 ± 4.13	0.045*	0.124	−0.56 (−1.12, 0.01)	0.052
Static	Frontal	Open	10.40 ± 6.10	12.10 ± 6.77	0.461	0.531	−0.23 (−0.79, 0.33)	0.009

*Note:* Values are presented as mean ± SD. Adjusted *p* values were obtained from ANCOVA controlling for age. Cohen's *d* values are reported with 95% confidence intervals. *p* < 0.05 was considered statistically significant. *significancy, *p* < 0.05.

**Table 4A:** Comparison of trunk–pelvic angular displacement velocity between high and low fear‐of‐pain groups according to Biodex level, kinematic plane, and visual condition.

Biodex	Plane	Eye	High fear (mean ± SD)	Low fear (mean ± SD)	*p* value (unadjusted)	*p* value (adjusted)	Cohen's *d* for unadjusted age (95% CI)	Partial eta squared for adjusted age
Level 5	Sagittal	Open	50.57 ± 42.50	38.60 ± 21.27	0.801	0.240	−0.49 (−1.05, 0.08)	0.039
Level 5	Sagittal	Closed	45.52 ± 36.83	37.89 ± 27.46	0.485	0.266	−0.44 (−1.00, 0.12)	0.012
Level 5	Transverse	Open	55.53 ± 37.05	55.53 ± 39.30	0.861	0.317	−0.46 (−1.02, 0.11)	0.029
Level 5	Transverse	Closed	51.85 ± 46.47	53.27 ± 41.05	0.771	0.597	−0.25 (−0.80, 0.31)	0.003
Level 5	Frontal	Open	116.20 ± 118.79	110.68 ± 127.68	0.252	0.668	0.04 (−0.52, 0.59)	0.002
Level 5	Frontal	Closed	109.20 ± 101.61	93.39 ± 76.96	0.534	0.574	−0.19 (−0.75, 0.37)	0.003
Level 2	Sagittal	Open	51.53 ± 30.38	44.88 ± 22.50	0.587	0.666	−0.16 (−0.71, 0.40)	0.003
Level 2	Sagittal	Closed	55.35 ± 51.46	37.81 ± 23.14	0.145	0.745	0.02 (−0.53, 0.58)	0.010
Level 2	Transverse	Open	50.67 ± 28.97	64.74 ± 43.00	0.303	0.671	−0.26 (−0.82, 0.30)	0.002
Level 2	Transverse	Closed	53.19 ± 42.88	46.65 ± 30.04	0.741	0.779	−0.20 (−0.76, 0.36)	0.00
Level 2	Frontal	Open	146.84 ± 133.95	95.11 ± 97.77	0.099	0.680	0.01 (−0.55, 0.56)	0.003
Level 2	Frontal	Closed	145.82 ± 289.25	78.28 ± 43.16	0.801	0.667	−0.26 (−0.82, 0.30)	0.002
Static	Sagittal	Open	20.77 ± 13.74	24.17 ± 23.08	0.573	0.135	−0.26 (−0.81, 0.30)	0.040
Static	Transverse	Open	28.09 ± 26.36	30.57 ± 32.29	0.712	0.163	−0.23 (−0.78, 0.33)	0.040
Static	Frontal	Open	50.31 ± 31.45	45.33 ± 33.43	0.426	0.298	−0.30 (−0.86, 0.26)	0.015

Abbreviations: CI, confidence interval; SD, standard deviation.

**Table 4B:** Comparison of lumbar–pelvic angular displacement velocity between high and low fear‐of‐pain groups according to Biodex level, kinematic plane, and visual condition.

Biodex	Plane	Eye	High fear (mean ± SD)	Low fear (mean ± SD)	*p* value (unadjusted)	*p* value (adjusted)	Cohen's *d* for unadjusted age (95% CI)	Partial eta squared for adjusted age
Level 5	Sagittal	Open	56.15 ± 40.01	46.79 ± 27.57	0.497	0.404	−0.38 (−0.93, 0.19)	0.011
Level 5	Sagittal	Closed	49.51 ± 35.52	54.79 ± 53.59	0.846	0.437	0.16 (−0.39, 0.71)	0.035
Level 5	Transverse	Open	74.41 ± 51.45	77.71 ± 53.49	> 0.999	0.507	−0.34 (−0.89, 0.23)	0.015
Level 5	Transverse	Closed	67.58 ± 58.16	74.98 ± 57.28	0.698	0.801	−0.15 (−0.71, 0.41)	0.000
Level 5	Frontal	Open	151.61 ± 147.20	153.42 ± 163.56	0.600	0.776	0.02 (−0.54, 0.57)	0.002
Level 5	Frontal	Closed	144.68 ± 109.28	127.28 ± 87.17	0.627	0.514	−0.26 (−0.82, 0.30)	0.003
Level 2	Sagittal	Open	56.45 ± 27.72	53.22 ± 42.84	0.372	0.740	0.01 (−0.54, 0.57)	0.005
Level 2	Sagittal	Closed	73.72 ± 93.33	45.31 ± 22.79	0.382	0.806	−0.02 (−0.58, 0.53)	0.007
Level 2	Transverse	Open	67.30 ± 40.10	90.49 ± 57.33	0.174	0.835	−0.18 (−0.74, 0.38)	0.00
Level 2	Transverse	Closed	71.01 ± 54.77	66.31 ± 43.06	0.907	0.936	−0.13 (−0.68, 0.43)	0.003
Level 2	Frontal	Open	182.81 ± 160.58	134.47 ± 118.07	0.229	0.852	−0.06 (−0.62, 0.49)	0.001
Level 2	Frontal	Closed	198.35 ± 364.48	133.16 ± 74.91	0.756	0.655	−0.28 (−0.83, 0.28)	0.001
Static	Sagittal	Open	27.40 ± 12.40	30.11 ± 25.17	0.573	0.146	−0.30 (−0.85, 0.26)	0.030
Static	Transverse	Open	36.75 ± 35.34	42.71 ± 43.24	0.497	0.242	−0.14 (−0.69, 0.42)	0.028
Static	Frontal	Open	78.77 ± 37.67	72.01 ± 45.19	0.372	0.26	−0.63 (−1.20, −0.06)	0.083

*Note:* Values are presented as mean ± SD. Adjusted *p* values were obtained after controlling for baseline age. Unadjusted *p* values were obtained using independent *t*‐tests or Mann–Whitney U tests, as appropriate. Cohen's *d* effect sizes are reported with 95% confidence intervals.

Table 1 indicated a significant difference in age between the two groups (*p *< 0.01). Therefore, group comparisons of the dependent variables were performed using mixed‐model analyses adjusted for age, kinematic plane, Biodex stability level, and visual condition (Table 2). The observed effect sizes were generally small, with the largest effect observed for trunk–pelvic angular displacement (*ηp* ^2 ^= 0.052), which approached the conventional threshold for a medium effect. The primary mixed‐model analysis revealed significantly greater trunk–pelvic angular displacement (*p* < 0.001) and significantly greater trunk–pelvic angular velocity in the high fear‐of‐pain group than in the low fear‐of‐pain group (*p* = 0.014).

However, in the stratified pairwise comparisons (Tables 4A and 4B), most differences did not reach statistical significance, likely reflecting reduced statistical precision after the sample was subdivided across multiple conditions.

Comparisons before and after adjustment for age (Tables 3A, 3B, 4A, and 4B) indicated that age influenced the observed between‐group differences in some subgroup analyses.

Before adjustment for age, nominally significant differences were observed under several conditions. In the static condition with eyes open, the high fear‐of‐pain group demonstrated greater transverse‐plane angular displacement in both the lumbar–pelvic (*p *= 0.045; Cohen's *d *= 0.56) and trunk–pelvic (*p *= 0.041; Cohen's *d *= 0.57) segments than the low‐fear group. Under Level 5 instability with eyes open, the high fear‐of‐pain group exhibited significantly greater frontal‐plane trunk–pelvic angular displacement (*p *= 0.005; Cohen's *d *= 0.63), as well as greater lumbar–pelvic (*p *= 0.015; Cohen's *d *= 0.43) and trunk–pelvic (*p *= 0.002; Cohen's *d *= 0.68) displacement. In addition, under Level 2 instability with eyes open, greater sagittal‐plane trunk–pelvic angular displacement was observed in the high fear‐of‐pain group (*p *= 0.010; Cohen's *d *= 0.45). These findings are reported in Tables 3A, 3B, 4A, and 4B.

After adjustment for age, significant between‐group differences were primarily retained in the transverse plane under unstable conditions. Specifically, the high fear‐of‐pain group (who were also older) demonstrated greater transverse‐plane angular displacement in both the lumbar–pelvic (Level 5: *p* = 0.007; *ηp* ^2 ^= 0.150; Level 2: *p* = 0.020; *ηp* ^2 ^= 0.115) and trunk–pelvic segments (Level 5: *p* = 0.005; *ηp* ^2 ^= 0.165; Level 2: *p* = 0.009; *ηp* ^2 ^= 0.141) during eyes‐open conditions. No other significant between‐group differences were observed in angular displacement or velocity across kinematic planes, Biodex levels, or visual conditions (Tables 3A, 3B, 4A, and 4B).

When visual input was removed (eyes closed), no significant between‐group differences were observed in maximum angular displacement at any stability level (Tables 3A, 3B, 4A, and 4B). Similarly, the angular velocity of both the trunk–pelvic and lumbar–pelvic segments did not differ significantly between groups across the perturbation levels or visual conditions.

Age was entered as a covariate in all models. The coefficient for age was significant for trunk–pelvic angular displacement (*β* = −0.116, SE = 0.033, *p* < 0.001), indicating that older age was associated with lower angular displacement. For the other outcomes, age did not significantly influence the results: lumbar–pelvic angular displacement (*β* = −0.004, SE = 0.036, *p* = 0.910), trunk–pelvic angular velocity (*β* = −0.460, SE = 0.314, *p* = 0.142), and lumbar–pelvic angular velocity (*β* = −0.309, SE = 0.394, *p* = 0.434). The partial eta squared for age as a covariate was *ηp* ^2 ^= 0.014 for trunk–pelvic angular displacement and negligible for the other outcomes (all *ηp* ^2 ^< 0.003).

### 3.1 Reliability Analysis

Intra‐session reliability during the first testing session demonstrated good‐to‐excellent consistency, with intraclass correlation coefficients (ICCs) ranging from 0.77 to 0.92. In the second session, ICC values ranged from 0.79 to 0.94. Inter‐session reliability also demonstrated good‐to‐excellent agreement, with ICC values ranging from 0.75 to 0.94.

Standard error of measurement (SEM) values ranged from 0.10 to 0.14, whereas minimal detectable change (MDC) values ranged from 0.20 to 0.28.

Similarly, postural stability‐related variables assessed under eyes‐closed conditions demonstrated high intra‐session and inter‐session repeatability. Intra‐session ICC values ranged from 0.78 to 0.95 in the first session and from 0.72 to 0.95 in the second session. Inter‐session ICC values ranged from 0.70 to 0.95. SEM values ranged from 0.11 to 0.14, and MDC values ranged from 0.22 to 0.28, indicating good measurement reliability across conditions.

## 4 Discussion

The primary finding of the present study was that individuals with CNSLBP who reported higher levels of fear of pain demonstrated greater angular displacement of both the trunk–pelvic and lumbar–pelvic segments in the transverse plane while standing on unstable surfaces under eyes‐open conditions. Importantly, these differences became evident only after adjustment for age, indicating that age influenced the observed between‐group differences in postural kinematics. No significant differences were observed in the sagittal or frontal planes after age adjustment, and the between‐group differences disappeared when visual input was removed. Because these subgroup analyses were exploratory and were not corrected for multiple comparisons, the findings should be interpreted cautiously and considered hypothesis‐generating.

The association between fear of pain and increased transverse‐plane displacement is consistent with contemporary biopsychosocial models of chronic low back pain, which emphasize that psychological factors can influence movement behavior and postural control. Recent evidence suggests that fear‐related cognitions may alter sensorimotor processing and movement strategies, leading to less efficient control of trunk motion during challenging balance tasks [33, 34]. Rather than simply reducing movement, individuals with elevated pain‐related fear may adopt alternative motor strategies characterized by increased variability and altered segmental coordination when exposed to postural threats or instability [35]. Recent systematic reviews have highlighted that psychological factors, including fear of movement and pain‐related anxiety, are associated with altered motor behavior and postural adaptations in people with chronic low back pain [36].

Interestingly, the observed differences were limited to the transverse plane. Rotational control of the trunk and lumbopelvic region requires precise coordination among the spinal stabilizing muscles, hip musculature, and sensorimotor feedback systems [37]. Recent studies suggest that rotational movements may be particularly sensitive to motor‐control deficits because they require greater intersegmental coordination than movements occurring primarily in the sagittal plane [38]. Impairments in trunk rotational control have been reported in individuals with chronic low back pain and may represent an early biomechanical manifestation of altered sensorimotor control [39, 40]. Therefore, the greater transverse‐plane displacement observed in the high‐fear group may reflect a reduced ability to regulate rotational motion when confronted with postural instability.

A notable finding was the influence of age on these associations. Before age adjustment, several apparent differences were observed across multiple planes and instability levels; however, most of these differences disappeared after adjustment, whereas the transverse‐plane findings remained significant. This pattern suggests that age contributed substantially to postural performance and may have obscured the specific association between fear of pain and kinematic behavior. Age‐related declines in proprioceptive acuity, reaction time, sensory integration, and neuromuscular control have been consistently reported and may influence trunk stability during balance tasks [41, 42]. The persistence of transverse‐plane differences after controlling for age suggests that these differences were not fully attributable to age alone. The exploratory subgroup analyses suggested additional differences in lumbar–pelvic kinematics under specific conditions (e.g., Level 5, transverse, eyes open). However, given the exploratory nature of these findings and the lack of adjustment for multiple comparisons, these results should be considered hypothesis‐generating rather than confirmatory. Future studies with larger samples and prespecified subgroup hypotheses are needed to validate these observations.

The effect sizes for the primary outcomes were generally small (*ηp* ^2 ^= 0.002–0.052), with the largest effect observed for trunk–pelvic angular displacement, indicating that although fear of pain was significantly associated with trunk–pelvic kinematics, the magnitude of these associations was modest. This suggests that other factors, such as age, pain intensity, or neuromuscular control, may also contribute to postural control deficits in individuals with CNSLBP. Age was a significant covariate only for trunk–pelvic angular displacement (*β* = −0.116, *p* < 0.001), indicating that age influenced this specific outcome and should be considered when interpreting the between‐group differences.

Another important observation was that the between‐group differences were present only under eyes‐open conditions and disappeared when visual input was removed. This finding suggests that visual information may play an important role in the expression of fear‐related motor adaptations. Individuals with chronic low back pain frequently demonstrate impairments in proprioceptive processing and lumbar position sense, which may result in greater reliance on visual feedback for postural regulation [43]. Recent evidence indicates that chronic pain can disrupt multisensory integration processes involved in balance control, leading individuals to rely more heavily on vision when maintaining stability [44]. When vision was removed, both groups may have experienced a similar reduction in postural control capacity, thereby masking the influence of fear of pain on movement behavior.

The primary mixed‐model analysis also demonstrated significantly greater trunk–pelvic angular velocity in the high‐fear group. However, this finding was not consistently observed in the exploratory stratified analyses. This discrepancy may reflect reduced statistical precision resulting from subdivision of the sample across multiple Biodex levels, movement planes, and visual conditions. Future studies with larger samples are needed to determine the specific contexts in which this association may be clinically meaningful.

Taken together, the findings suggest that fear of pain may influence postural control in CNSLBP primarily through alterations in rotational movement control during visually guided balance tasks performed on unstable surfaces. The results further indicate that age should be considered an important covariate when investigating the relationship between psychological factors and movement behavior in chronic low back pain populations. Future prospective studies with larger samples, adjustment for multiple testing, and comprehensive assessments of sensorimotor function are needed to confirm these findings and clarify the mechanisms linking fear of pain, aging, and postural control.

## 5 Limitations

Like other studies, the present study has several limitations. Participants had CNSLBP for at least 12 months before testing and varied in their level of physical activity. The duration of chronicity and physical activity level may influence disability and psychological factors in these individuals. Future studies should consider more homogeneous samples with respect to the duration of LBP and physical activity level. Muscle activity should also be assessed alongside kinematic measures during postural perturbations. The initial sample‐size estimation was based on a small pilot study, which may have overestimated the true effect size. The final sample included 24 participants in the high‐fear group rather than the prespecified target of 25, which may have limited statistical precision. Therefore, larger confirmatory studies are recommended. Age was a significant covariate only for trunk–pelvic angular displacement (*β* = −0.116, *p* < 0.001), highlighting the importance of age adjustment in future studies.

Another limitation is the lack of correction for multiple comparisons in the exploratory subgroup analyses (Tables 3A, 3B, 4A, and 4B), which may have increased the risk of Type I error. These findings were therefore interpreted conservatively and emphasized as hypothesis‐generating. Additionally, the cross‐sectional design precludes conclusions regarding causality. Finally, the specific characteristics of the study sample, including relatively mild pain intensity at rest (VAS < 3), may limit the generalizability of the findings to individuals with more severe pain or different clinical presentations of chronic low back pain.

## 6 Implications for Physiotherapy Practice

The findings of this study emphasize the importance of considering psychological factors, particularly fear of pain, in the assessment and rehabilitation of individuals with chronic non‐specific low back pain (CNSLBP). Exploratory analyses indicated that participants with higher fear of pain demonstrated greater trunk–pelvic and lumbar–pelvic angular displacement in the transverse plane during visually guided balance tasks on unstable surfaces, suggesting altered movement coordination under challenging conditions.

These findings support the integration of routine assessment of pain‐related fear within a biopsychosocial framework. Rehabilitation should address not only physical impairments but also maladaptive pain beliefs and fear‐related behaviors through approaches such as pain neuroscience education, graded exposure, and exercise therapy.

Additionally, interventions targeting trunk rotational control, sensorimotor function, and dynamic balance may be beneficial. Given the influence of visual input and age on movement performance, rehabilitation programs should also consider sensory integration and age‐related adaptations to optimize functional outcomes.

## Author Contributions

**Mohammad Reza Salehifar:** conceptualization, investigation, data curation. **Rozita Hedayati:** conceptualization, methodology, writing – review and editing, project administration. **Marzieh Mortezanejad:** methodology, writing – original draft, project administration. **Rasool Bagheri:** supervision, writing – review and editing, software. **Fatemeh Paknazar:** formal analysis, writing – review and editing, data curation.

## Funding

The authors have nothing to report.

## Ethics Statement

This study was approved by the Ethics Committee of Semnan University of Medical Sciences and Health Services (ID: IR. SEMUM.REC.1398.227).

## Consent

All participants provided written informed consent before participating in the study.

## Conflicts of Interest

The authors declare no conflicts of interest.

## Transparency Statement

Rozita Hedayati affirms that this manuscript is an honest, accurate, and transparent account of the study being reported; that no important aspects of the study have been omitted; and that any discrepancies from the study as planned (and, if relevant, registered) have been explained.

## Data Availability

Data are available from the corresponding author upon reasonable request.
